# Ambulatory (24 h) blood pressure and arterial stiffness measurement in Marfan syndrome patients: a case control feasibility and pilot study

**DOI:** 10.1186/s12872-016-0263-x

**Published:** 2016-05-06

**Authors:** Matthias Hillebrand, Ghazaleh Nouri, Bernhard Hametner, Stephanie Parragh, Jelena Köster, Kai Mortensen, Achim Schwarz, Yskert von Kodolitsch, Siegfried Wassertheurer

**Affiliations:** Universitäres Herzzentrum Hamburg, Universitätskrankenhaus Hamburg-Eppendorf, Martinistrasse 52, 20246 Hamburg, Germany; Universitätsklinikum Schleswig-Holstein, Medizinische Klinik II, Ratzeburger Allee 160, 23538 Lübeck, Germany; AIT Austrian Institute of Technology, Donau-City Str. 1, 1220 Vienna, Austria; IEM GmbH, Cockerillstr. 69, 52222 Stolberg, Germany

## Abstract

**Background:**

The aim of this work is the investigation of measures of ambulatory brachial and aortic blood pressure and indices of arterial stiffness and aortic wave reflection in Marfan patients.

**Methods:**

A case-control study was conducted including patients with diagnosed Marfan syndrome following Ghent2 nosology and healthy controls matched for sex, age and daytime brachial systolic blood pressure. For each subject a 24 h ambulatory blood pressure and 24 h pulse wave analysis measurement was performed.

**Results:**

All parameters showed a circadian pattern whereby pressure dipping was more pronounced in Marfan patients. During daytime only Marfan patients with aortic root surgery showed increased pulse wave velocity. In contrast, various nighttime measurements, wave reflection determinants and circadian patterns showed a significant difference.

**Conclusions:**

The findings of our study provide evidence that ambulatory measurement of arterial stiffness parameters is feasible and that these determinants are significantly different in Marfan syndrome patients compared to controls in particular at nighttime. Further investigation is therefore indicated.

## Background

Marfan syndrome is connected with several disorders including alterations of the cardiovascular system, affecting especially the proximal aorta. Changes of the aortic diameter have been identified in patients with Marfan syndrome [1–4]. Clinic brachial blood pressure measurements might be unable to reflect these changes accordingly. Additionally, during the last decades non-invasive methods to assess aortic stiffness and wave reflections have evolved which potentially offer a deeper insight in the mechanisms of the cardiovascular system and especially in aortic properties [5]. However, in patients with Marfan syndrome only a very limited number of such studies are described in literature. These studies include only small numbers of patients in different stages of the disease and several studies are lacking a control group [1–3, 6–12].

In recent years new techniques to perform pulse wave analysis have emerged where a common cuff is used to measure pulse waves. In combination with mathematical models and algorithms, this enables operator-independent and automated quantification of central waveforms and its dependent pulse wave parameters, also in larger cohorts [13–16]. In combination with an ambulatory blood pressure measurement device, 24 h ambulatory pulse wave analysis also seems to be possible.

The aim of this study is to perform a pilot study based on ambulatory 24 h blood pressure and pulse wave analysis in patients with Marfan syndrome compared with a control group to investigate feasibility and to analyze potential diurnal variation of several parameters reflecting the status of the cardiovascular system, especially by quantifying wave reflections and arterial stiffness.

## Methods

Thirty patients with diagnosed Marfan syndrome following Ghent2 nosology [17] were enrolled in the study. Two patients had to be excluded due to incomplete data recording and one patient due to unstable sinus rhythm. The remaining 27 patients were matched with 27 healthy subjects from the Lübeck standard collective. Matching criteria were sex, age and daytime peripheral systolic blood pressure readings. For further subgroup analysis the Marfan group was divided into 13 patients with and 14 patients without aortic root surgery. For each subject, an ambulatory (24 h) blood pressure and pulse wave measurement series was taken using the Mobil-O-Graph device (IEM, Stolberg, Germany) with inbuilt ARCSolver pulse wave analysis algorithms (AIT Austrian Institute of Technology GmbH, Vienna, Austria). For the analysis, ARCSolver version 1.6.3 was applied using mean arterial and diastolic blood pressure for central waveform calibration [18]. The daytime period was defined from 9 am to 9 pm and the nighttime period from 1 am to 6 am. During daytime 15 min intervals and during nighttime 30 min intervals have been programmed. Only measurements with PWA quality index 1 and 2 were included in the study.

For the comparison of day- and nighttime, all measurements taken within the corresponding time interval were averaged per person to obtain 1 day and one night value per person. Mean values from both Marfan and control groups were calculated from these values and tested for significant differences by means of a two-sided Student t-test (equal variances) or a Welch test (unequal variances) after checking for normality with the Kolmogorov-Smirnoff test. Differences between more than two groups were assessed with the Kruskal-Wallis-test. The Runs test was used to determine the existence of diurnal profiles. *P* < 0.05 was considered significant for all tests.

For graphical presentation, all measurements within 1 h were averaged per person and then per group and a 95 % confidence interval was calculated. Solely for the purpose of plotting, the resulting 24 values were smoothed using a 3-h moving average.

Various hemodynamic parameters are given by the Mobil-O-Graph and its inbuilt ARCSolver which have been described in previous publications. Therefore only a short description of the measuring process and the derived parameters used in this study will be presented here.

The Mobil-O-Graph is a validated oscillometric ambulatory blood pressure measurement device [19]. After performing the blood pressure measurement, the cuff is inflated to the brachial diastolic pressure level (pDBP) and the oscillations (pulse waves) are recorded for 10 s. After the 24 h measurement circle, all measurements are transferred to the HMS client software and analyzed with the ARCSolver algorithms, which have been validated in invasive and non-invasive studies [13, 14]. From the estimated central curve, again systolic (cSBP), diastolic (cDBP) and pulse pressure (cPP) can be directly calculated. Furthermore, indices of central hemodynamics, like augmentation index (AIx) and subsequently AIx75, which should represent the augmentation index at a heart rate of 75 beats per minute [5], can be computed. To obtain more enhanced wave reflection parameters, a blood flow model is used to calculate characteristic impedance which enables wave separation analysis [20–23]. Thereby, the central pressure curve is separated in the forward (Pf) and backward (Pb) travelling wave. The ratio of their amplitudes is denoted as reflection magnitude (RM). Additionally an estimated aortic pulse wave velocity (PWV) based on age, central pressure and characteristic impedance can be calculated [24, 25].

## Results

In the Marfan syndrome as well as in the control group, 14 female and 13 male subjects were included. There are no significant differences in age and weight, but the Marfan patients are on average 12 cm taller. Of the 27 patients with Marfan syndrome, 13 underwent aortic root surgery (ARS), 14 (9 ARS) are taking beta blockers and 10 (6 ARS) are treated with ACE inhibitors, CCB or ARB. Controls were free of drugs. During daytime on average 25 valid measurements for the Marfan group and 26 for the control group were analyzed, during nighttime 7 and 9, respectively. The baseline characteristics of both groups are summarized in Table 1. A comparison of the hemodynamic parameters between Marfan patients and controls is given in Table 2 for day- and nighttime separately.

**Table 1:** Baseline characteristics

Parameters	Marfan	Controls	*P*-Value
Patients	27	27	
Men/Women	13/14	13/14	
Age (years)	38.9 (11.0 SD)	39.2 (12.3 SD)	0.92
Weight (kg)	78.4 (17.7 SD)	75.0 (12.2 SD)	0.42
Height (cm)	190 (18 SD)	174 (10 SD)	<0.001
Beta blocker n (%)	14 (52 %)	n.a.	
ARB/CCB/ACE inhibitors n (%)	10 (37 %)	n.a.	
Aortic root surgery	13 (48 %)	n.a.	

Results are given as mean (SD); *ARB* angiotensin receptor blocker, *CCB* calcium channel blocker, *ACE* angiotensin-converting-enzyme

**Table 2:** Comparison day and night

	Daytime		Nighttime	
Parameter	Marfan	Controls	Marfan	Controls
Heart rate (bpm)	**76.6 (10.4 SD)**	**73.6 (9.20 SD)**	**63.9 (11.2 SD)**	**60.0 (8.30 SD)**
DBP brachial (mmHg)	**78.2 (7.26 SD)**	**75.9 (7.09 SD)**	**61.2 (7.72 SD)**	**61.2 (7.35 SD)**
SBP brachial (mmHg)	**119 (9.04 SD)**	**119 (8.54 SD)**	**100 (10.8 SD)**	**104 (9.88 SD)**
PP brachial (mmHg)	40.3 (6.29 SD)	42.6 (5.46 SD)	38.9 (6.12 SD)^*^	42.5 (5.90 SD)^*^
DBP central (mmHg)	**79.7 (7.32 SD)**	**77.3 (7.24 SD)**	**62.5 (7.6 SD)**	**63.0 (7.81 SD)**
SBP central (mmHg)	**119 (9.22 SD)**	119 (10.1 SD)	**108 (11.7 SD)** ^*^	116 (14.6 SD)^*^
PP central (mmHg)	**39.3 (7.73 SD)**	**42.1 (7.6 SD)**	**45.7 (8.50 SD)** ^*^	**53.0 (11.4 SD)** ^*^
Pf (mmHg)	**26.2 (4.55 SD)**	**27.8 (5.57 SD)**	**28.8 (4.84 SD)** ^*^	**33.3 (8.49 SD)** ^*^
Pb (mmHg)	**15.2 (3.57 SD)**	**16.3 (3.3 SD)**	**19.2 (4.39 SD)** ^*^	**22.7 (4.73 SD)** ^*^
RM (−)	**57.8 (6.23 SD)**	**58.6 (7.54 SD)**	**66.6 (7.76 SD)**	**68.7 (6.13 SD)**
PWV (m/s)	**6.55 (1.14 SD)**	6.67 (1.19 SD)	**6.15 (1.04 SD)**	6.53 (1.2 SD)
AP (mmHg)	**8.13 (3.40 SD)**	**8.61 (4.53 SD)**	**13.3 (7.47 SD)**	**14.2 (7.48 SD)**
AIx (−)	**19.4 (6.20 SD)**	**19.4 (8.20 SD)**	**26.0 (10.9 SD)**	**25.0 (12.5 SD)**

Results are given as mean (SD). *SBP* systolic blood pressure, *DBP* diastolic blood pressure, *PP* pulse pressure, *Pf(b)* amplitude of forward (backward) travelling pressure wave, *RM* reflection magnitude, *PWV* pulse wave velocity, *AP* augmented pressure, *AIx* augmentation index. ^*^ *P* < 0.05 Marfan vs. controls. Bold: *P* < 0.05 day vs. night

During daytime, no significant differences in pSBP, pDBP, pPP and heart rate (HR) can be detected, although pPP is slightly lower and HR is slightly higher in the Marfan group. A diurnal profile for HR is provided by Fig. 4. Also for the corresponding central values cSBP, cDBP and cPP no significant differences can be seen during daytime.

**Fig. 1:**
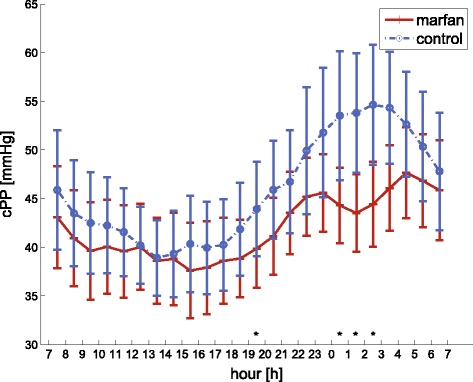
Comparison of the diurnal rhythm in central pulse pressure for Marfan patients (*solid red line*) and controls (*dashed blue line*) over 24 h. Error bars are 95 % convidence intervals. * indicates a significant difference, *P* < 0.05

During nighttime, brachial systolic pressure levels are markedly lower than during daytime. This dipping effect is slightly, but not significantly, more pronounced in the Marfan group. Notwithstanding, this leads to a significant difference between groups for pPP during nighttime (38.9 mmHg vs 42.5 mmHg, *p* = 0.03). Beyond these brachial differences, significant differences can be seen for central systolic and pulse pressure values as well as for forward and backward wave amplitudes during the night, as shown in Fig. 1 and Table 2. To quantify the pressure dipping, the ratio of daytime cSBP and nighttime cSBP was computed for Marfan and controls respectively (1.11 vs 1.04, *p* = 0.02).

**Fig. 2:**
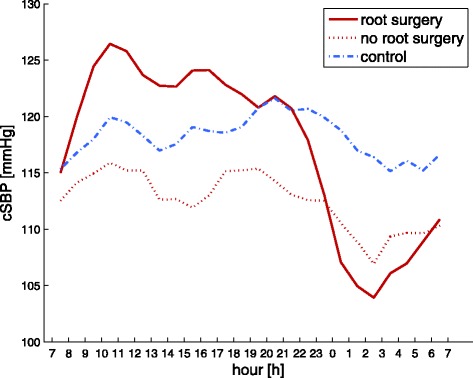
Comparison of the diurnal rhythm in central systolic pressure for Marfan patients with (*solid red line*) and without root surgery (*dotted red line*) and controls (*dashed blue line*) over 24 h

In subgroup analysis (see also Table 3), the effects described above are most dominant for Marfan patients who underwent aortic root surgery (*n* = 13, 8/5 m/w). During daytime, central systolic pressure is higher for patients with root surgery than for the remaining Marfan group as well as for the control group, whereas during nighttime, the opposite can be observed as illustrated in Fig. 2. Comparison of the dipping effect between day and night, i.e. the ratio of cSBP, in all three groups using the Kruskal-Wallis test therefore shows a significant difference between the three groups (*p* = 0.01). In post-hoc analysis, the dipping observed in patients who underwent root surgery is significantly stronger than for the remaining Marfan patients (1.12 vs. 1.04, *p* < 0.05) as well as controls (1.12 vs. 1.04, *p* < 0.05). The difference between Marfan patients without root surgery and controls is non-significant.

**Table 3:** Comparison day and night in subgroup analysis of Marfan patients

	Daytime		Nighttime	
Parameter	Root surgery	No root surgery	Root surgery	No root surgery
Heart rate (bpm)	**73.0 (9.55 SD)**	**80.0 (10.3 SD)**	**65.1 (8.81 SD)**	**62.7 (13.3 SD)**
DBP brachial (mmHg)	**79.4 (6.95 SD)**	**77.1 (7.61 SD)**	**60.7 (8.70 SD)**	**61.7 (6.98 SD)**
SBP brachial (mmHg)	**121 (7.08 SD)**	**116 (10.2 SD)**	**99.0 (10.4 SD)**	**101 (11.5 SD)**
PP brachial (mmHg)	**41.8 (6.39 SD)**	39.0 (6.10 SD)	**38.4 (4.86 SD)**	39.4 (7.25 SD)
DBP central (mmHg)	**81.1 (7.09 SD)**	**78.3 (7.54 SD)**	**61.8 (8.58 SD)**	**63.1 (6.83 SD)**
SBP central (mmHg)	**123 (7.04 SD)** ^*^	115 (9.43 SD)^*^	**105 (11.2 SD)**	111 (12.0 SD)
PP central (mmHg)	42.2 (7.27 SD)	**36.6 (7.38 SD)**	43.8 (6.73 SD)	**47.5 (9.77 SD)**
Pf (mmHg)	27.7 (4.56 SD)	**24.7 (4.17 SD)**	27.7 (4.66 SD)	**29.7 (4.96 SD)**
Pb (mmHg)	16.6 (3.18 SD)	**14.0 (3.55 SD)**	18.3 (3.23 SD)	**20.1 (5.21 SD)**
RM (−)	**59.8 (5.78 SD)**	**56.0 (6.27 SD)**	**66.4 (7.28 SD)**	**66.8 (8.45 SD)**
PWV (m/s)	**7.09 (1.34 SD)** ^*^	**6.06 (0.641 SD)** ^*^	**6.47 (1.25 SD)**	**5.86 (0.731 SD)**
AP (mmHg)	**8.90 (3.98 SD)**	**7.42 (2.71 SD)**	**12.6 (6.63 SD)**	**14.0 (8.37 SD)**
AIx (−)	**19.9 (8.38 SD)**	**18.9 (3.41 SD)**	**26.2 (11.3 SD)**	**25.8 (10.9 SD)**

Results are given as mean (SD). *SBP* systolic blood pressure, *DBP* diastolic blood pressure, *PP* pulse pressure, *Pf(b)* amplitude of forward (backward) travelling pressure wave, *RM* reflection magnitude, *PWV* pulse wave velocity, *AP* augmented pressure, *AIx* augmentation index. ^*^ *P* < 0.05 root surgery vs. no root surgery. Bold: *P* < 0.05 day vs. night

Amplitudes of forward and backward travelling waves show no significant differences during daytime, but higher differences during nighttime, both being smaller for the Marfan group, leading to a significant mean difference in backward wave amplitude (3.5 mmHg, *p* = 0.008). The subgroup analysis shows that reflected waves are higher in Marfan patients with root surgery compared to those without at daytime (*p* = 0.05) but lower at nighttime although non-significant.

The resulting differences in central hemodynamics between the two Marfan groups are finally manifested in significantly different circadian patterns assessed by the means of a Runs test (*p* < 0.05) as exemplarily visualized by Fig. 2.

**Fig. 3:**
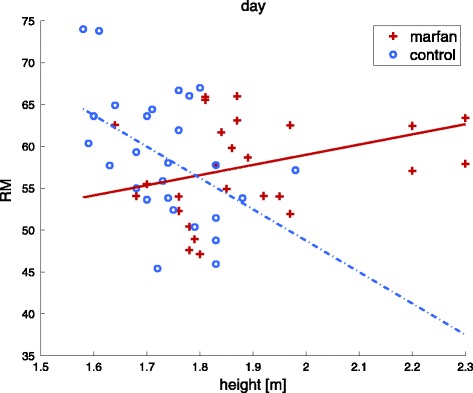
Scatter plot with regression lines for the daytime reflection magnitude with respect to height for the Marfan and control group separately. Nighttime plots are confirmatory

Reflection magnitude RM, as the ratio of backward and forward wave amplitudes, is slightly lower for the Marfan group during nighttime. Nevertheless, RM as well as AIx and AIx75 do not show any statistically significant differences between the two groups neither during daytime nor nighttime. An analysis of covariates unveils a negative relation between body height and all wave reflection parameters (RM, AIx, AIx75 and augmentation pressure) for the control group, while this effect is not present for the group of Marfan patients, as shown in Fig. 3 for daytime RM (regression slopes: 12.2 vs −37.5, *p* < 0.01, Marfan vs. controls). Subgroup analysis shows no relevant difference in slopes for both Marfan subgroups. This observation also remains existent at nighttime.

Pulse wave velocity is similar between Marfan patients and controls. However, we found PWV to be higher in Marfan patients with root surgery compared to Marfan patients without (7.1 m/s vs. 6.1 m/s, *p* = 0.01, during daytime and 6.5 m/s vs. 5.9 m/s, *p* = 0.13, during nighttime). Of note, the pressure levels for the Marfan subgroup with ARS are lower than for those without ARS.

## Discussion

To our knowledge, this is the first study comparing patients with Marfan syndrome and healthy controls by means of a 24 h ambulatory blood pressure measurement in combination with a 24 h ambulatory pulse wave analysis.

In general, all parameters under investigation showed a very similar behavior during daytime for both groups.

In contrast, pronounced differences could be found during nighttime. The Marfan cohort showed a significantly increased aortic systolic dipping compared to controls. Furthermore, aortic pulse pressure was significantly lower for Marfan patients. The physiological link for this observation could be wave reflections, in our case measured by the amplitude of the backward travelling waves Pb, which showed significant differences. This parameter has already demonstrated its distinct role as an independent predictor of cardiovascular events in studies for both high-risk [21] as well as population based cohorts [26, 27] beyond brachial pressures.

At first glance, the cause for the similar peripheral pressure levels in both groups might be attributed solely to the antihypertensive medication. However, wave reflections are also a major determinant of pulse pressure and alterations in aortic wave reflection are only partially visible in traditional peripheral pressure readings. Therefore, dedicated analysis of aortic hemodynamics potentially promises additional insights.

Indeed there is an obvious difference in diurnal patterns for aortic hemodynamics between controls and Marfan patients with and without aortic root surgery when recalling Fig. 2. An important determinant of these different patterns may be identified by the circadian behavior of the forward pressure wave. Controls seem to react on the loss of gravitational influence caused by the change in body position at nighttime with an increase in Pf and cPP. In contrast, changes in the magnitude of Pf between day- and nighttime are far less pronounced within the Marfan group and subgroup analysis shows no change at all for patients who underwent aortic root surgery (Table 3). For patients without aortic root surgery, Pf is continuously lower than for controls, which might indicate a reduced hemodynamic compensation compared to controls at nighttime. An increase in aortic diameter potentially masks early impairment of the arterial wall and leads to an additional aortic ‘pseudo’ volume and altered vessel tapering respectively geometry. Subsequent stiffening of the microcirculation may furthermore affect the feedback potential of the Nervus vagus and may additionally influence venous return at nighttime. A consequence may be manifested in reduced pulse pressures and diminishing effects of body height on arterial wave reflection as already reported earlier by Segers et al. [1]; compare Fig. 3.

In addition, Pf is positively associated with pulse wave velocity. This known link is consistently reflected in our data. Analogous to observed forward pressure wave amplitudes, PWV is significantly higher in patients with root surgery compared to non-root surgery patients during daytime. In contrast, non-root surgery group PWV is always below the controls. Altogether this indicates that PWV is affected by structural properties of the arterial tree as well as cardiac performance. Major physical determinants of arterial stiffness are vessel diameter and progress of impaired vascular function. Both factors are significantly influenced by the pathogenesis of Marfan’s disease and are therefore a likely explanation for the findings of our study.

With respect to earlier research in this domain the results from this study are generally similar to previous findings although case control studies applying PWA and/or ABPM in patients with Marfan syndrome are rare.

Jondeau et al. investigated the role of peripheral and central pulse pressure as a determinant of ascending aorta dilation in patients with Marfan syndrome [2]. They found that carotid pulse pressure is a major determinant, whereas brachial pulse pressure is not. Additionally their data indicates a slightly lower carotid pulse pressure in the Marfan group compared to controls while they report the same brachial pulse pressure. This is in line with our findings especially during the night, where the pulse pressure amplification is somewhat higher in the Marfan group leading to statistically significant lower values in cPP, Pf and Pb. In both studies, the heart rate in the Marfan group is slightly elevated (3-4 bpm), which is often seen related with higher pulse pressure amplification. Segers et al. as well as Payne et al. do not report significant blood pressure differences in their case-control studies at any location and they give slightly lower heart rates for their Marfan groups [1, 12]. However, results are not fully comparable since cardiac medication and especially the use of beta blockers, which are directly affecting heart rate, differed between the respective study populations; medication was either withheld for 24 h [2] or 48 h [12] prior to examination or was not specified [1].

Reflection magnitude is not significantly different between the groups in our study, which is in line with the results obtained by Segers et al. The fact that RM is comparable between the patient groups in the present analysis, even though Pb significantly differs during nighttime, may be explained by the more pronounced dipping, differences in heart rate and the therefore generally lower central pulse pressure levels in the Marfan group.

The augmentation index as well as AIx75 was not found to be different between the groups in our study, which is again in line with findings in [1]. In contrast, AIx was higher in the Marfan group in the study of Payne et al. [12]. Comparisons between these studies need particular caution. While our study used calculated central pressure curves by a transfer function from brachial readings, the two other studies used carotid pressure waves as surrogates for central pressure waves. The augmentation index is influenced by cardiac properties and heart rate. Consequently amount and type of medication, which influence certain parts of the cardiovascular system differently, should be integrated in the interpretation, not only for AIX but for all pulse wave parameters [6, 28].

Pulse wave velocity is fairly similar between Marfan and controls in our study which corresponds to findings by Segers et al. and Payne et al. who report no significant differences between the groups. Nevertheless in both studies PWV is slightly elevated in the Marfan group and even significantly higher in a study from Hirata et al. [7]. Vitarelli et al. found significantly elevated PWV compared to controls in Marfan patients with aortic dilation but not in Marfan patients with normal aortic diameters [10]. Patients with Marfan syndrome suffer from a gradual progression in aortic root disease and thus do not represent a uniform collective. This could explain different findings in the different studies. A study by Mortensen et al. divided Marfan patients in 2 groups depending on progression of aortic root disease. While peripheral blood pressure levels were similar in both groups, the group with progression of aortic root disease had a higher pulse wave velocity. These observations from Vitarelli et al. as well as Mortensen et al. are in line with our findings during daytime when dividing the Marfan group into patients with and without aortic root surgery.

From a clinical point of view the findings of this pilot trial unveil pathways for future research as well as potential clinical utility. Our study is the first to apply ambulatory 24-h-blood pressure and arterial stiffness measurements in Marfan syndrome. Our cohort was small and differences had to be pronounced to reach statistical significance. Our findings show that circadian blood pressure profiles are significantly different in Marfan patients compared to controls. This observation appears to have clinical significance as it is well documented that the risk for aortic dissections is lowest at 4 am in the morning and highest between 8 and 11 am [29]. Our measurements of central systolic blood-pressure recapitulate this peak with highest pressures obtained between 7 and 11 am in the morning. Interestingly, these peaks were most pronounced in patients after aortic root surgery, which underpins the need for medications that manage these pressure peaks in postsurgical Marfan syndrome patients.

**Fig. 4:**
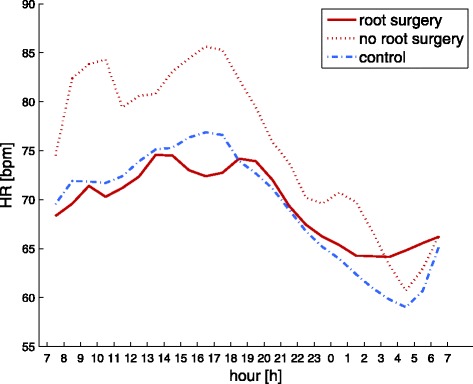
Comparison of the diurnal rhythm in heart rate for Marfan patients with (*solid red line*) and without root surgery (*dotted red line*) and controls (*dashed blue line*) over 24 h

Within our cohort only low doses of beta blockers have been prescribed which may also explain the observed heart rates in our study. In a recent study of children with Williams-Beuren syndrome who had borderline hypertensive peripheral blood pressures without prescription of beta blockers increased heart rates at nighttime as an early hallmark of cardiovascular changes have been reported [30]. With respect to the diurnal heart rate patterns Fig. 4 unveils again particular differences for the aortic root surgery group in the morning and forenoon.

The origin of the observed behavior may be solely owed to the slightly increased beta blocker prescription in this group. Nevertheless in a former study of Marfan patients we observed increased AIx75 in persons after conduit operations compared to those without aortic surgery [4]. Therefore we speculate that disease progression and the loss of conduit artery compliance, of course finally by aortic grafting, may overstrain the micro vascular and vagal system to manage orthostatic effects properly. Although venous return to the left ventricle is increased in supine position our Marfan syndrome patients seem to have impaired ventricular-vascular coupling performance to cope with such conditions. This is indicated by significantly decreased preload dependent parameters, in particular amplitudes of the forward and subsequent backward pressure wave as well as pulse pressure, in our Marfan syndrome patients at nighttime but not at daytime compared to controls. This leads to the paradox observation that within our Marfan syndrome cohort nighttime systolic but not diastolic pressure dipping effects are more pronounced compared to controls which is typically associated with better outcome. A similar mirage is well known from patients with reduced left ventricular ejection fraction where low values of systolic and pulse pressures are associated with higher risk [31, 32] and therefore show an inverted behaviour compared to traditional risk patterns. We therefore speculate that this paradox observation may have impact on future risk stratification in Marfan syndrome patients but further research is warranted.

Of note, symptoms like fatigue, dizziness and orthostatic symptoms limit the use, and dosage of medications in Marfan syndrome patients. 24-h central blood-pressure profiles with marked decreases during the night may be considered for timing of medications in future for potentially personalized therapy regimes.

## Limitations

The interpretation of the presented results needs to be done considering certain limitations. The design of our study is cross sectional and therefore no predictions but associations can be made. Furthermore, the effective influence of drugs remains open as treatment was not withheld during the study period. Sample size and available patient characteristics are always limited in such special cohorts. However, ambulatory recordings reduce measurement variability significantly, increase statistical power and therefore strengthen the presented results, also in the context of earlier work within the domain.

## Conclusion

In contrast to daytime measurements we found significant differences among patients and controls at nighttime for brachial pulse pressure and for central hemodynamics. In particular, aortic systolic dipping and altered aortic wave reflection lead to a significantly modified diurnal pattern in Marfan syndrome patients compared to controls. Assessment of ambulatory measurements is feasible but further investigations are indicated.

### Ethics, consent and permissions

The study was approved by the dedicated ethics committee of Universitäres Herzzentrum Hamburg as well as Universitätsklinikum Schleswig-Holstein. All participants provided written informed consent; no children were included.

### Consent for publication

N/A.

### Availability of data and materials

Raw data supporting the obtained results can be requested from the corresponding author.

## Abbreviations

ABPM: ambulatory (24 h) blood pressure measurement
ACE: angiotensin-converting-enzyme
AIx: augmentation index
AIx75: augmentation index normalized to a heart rate of 75 bpm
AP: augmentation pressure
ARB: angiotensin receptor blocker
ARS: aortic root surgery
CCB: calcium channel blocker
cDBP: central diastolic blood pressure
cPP: central pulse pressure
cSBP: central systolic blood pressure
HR: heart rate
Pb: amplitude of backward travelling wave
pDBP: peripheral diastolic blood pressure
Pf: amplitude of forward travelling wave
pPP: peripheral pulse pressure
pSBP: peripheral systolic blood pressure
PWA: pulse wave analysis
PWV: pulse wave velocity
RM: reflection magnitude
